# Gleanings

**Published:** 1894-04

**Authors:** F. H. Lutze

**Affiliations:** Brooklyn, N. Y.


### GLEANINGS.

F. H. Lutze, M. D., Brooklyn, N. Y.

Ovary, right, sensitive to touch and pressure. Apis.

Vaginismus, from excessive coitus. Arnic.

Ovaralgia worse on slight motion, yet cannot lie still, though motion increases the pain; constant moaning, shooting from left to right. Bry.

Hæmorrhage profuse and painless, bright red, worse from motion produced by excitement. Calc-c.

Pregnancy during, heart-burn on going to bed at night. Conium.

— aching pains every night after going to bed, better from getting up and moving about. Conium.

Labor, after colicy bearing-down pain, each accompanied with a gush of blood which causes relief. Cyclam.

Hæmorrhage active, with nausea, like the stream from a pump; with every pulsation of the heart there is a peculiar gush; blood does not easily coagulate and is bright red. Ipecac.

Menses with intermittent flow; profuse, clotted, with severe headache before and during the menses; often with nausea and vomiting. Kreos.

Neuralgia, uterine, grinding pains in uterus or rectum cause her to scream; suffers several days before discharge, improves but gets worse again. Nux-v.

Hæmorrhage from recent frights. Opi.

Milk in breasts instead of menses. Phos., Rhus (Merc.).

— — — after the menses. Cyclam.

Pregnancy, pain in right groin preventing motion in latter months of pregnancy. Podoph.

Menorrhagia better from exercise of walking. Sabina.

Confinement after anus prolapsed. Ruta.

Dysuria from reflex bladder trouble from uterine irritation, prolapse or flexion, distress at neck of bladder, burning pain, sleeplessness. Senecio.

Pregnancy, and in children, excessive straining to stool, stool covered with mucus; discharge bloody, slow, and difficult even of soft stool; stool hard, like sheep's dung; sleeplessness; insufficient stool. Sepia.

Menses every two to three months with repeated paroxysms of icy coldness of whole body. Silicea.

Pregnancy, during, cannot drink water for the sight of it causes nausea and vomiting. Bryonia.

### Coryza.

Air, sensitive even to warm air of room. Rumex.

— worse only from cold, damp air out of doors. Kali-bi., Phos., Dulc.

— seems to penetrate into the skull and brain, causing a cold sensation there. Cimicifuga.

Dry stuffed feeling in nose, yet a constant discharge, excoriating nostrils and upper lip; worse left nostril. Arum-tri. (Nitric-acid).

Coryza, discharge excoriates upper lip. Cepa.

— worse P. M.; better A. M. Sticta.

— — — alæ and columnæ nasi. Merc-v.

Stuffed feeling at root of nose, coryza dry at night, fluent by day; worse three A. M.; worse left nostril. Nux-v.

### Larynx.

Inflammation of larynx, with violent palpitation of heart, so as to cause suffocation. Guajacum.

Laryngismus stridulus. Chlorinæ aqua $3^x$ for relief.

— — if glands are swollen. Calc-iod.

Croup, rattling breathing, as if next cough would bring up large quantities of mucus; but cough is dry, hard; no fever; cool, sweaty skin. Bromium.

### Cough.

Cough after a short nap. Aral-rac.

— during day, none at night after lying down. Rumex, Thuja.

— — — loose, dry at night. Euphras.

— — — — dry, with stitches in the right side, with severe hoarseness every evening at five P. M., so that the voice could scarce be heard. Chelidon.

Cough from least current of air, as even of any one passing by. Calc-c.

— — want of breath. Aurum.

— spasmodic, excited by tickling in the pharynx and roof of mouth. Lactuca-vir.

— when spoken to. Ars.

— with bursting pain in occiput, loss of taste and smell, and redness of tip of nose, soreness through lower part of abdomen, holds abdomen with hand on coughing, cutting pain in right side of throat which aches afterward. Calc-c.

— worse after eating; worse trying to suppress it. Acon. (Ignat.), Marum.

— with stitches under left false ribs, stitches there also from breathing, worse lying on the painless side. Acon.

— a long attack mornings, dry, ends by raising a little white mucus, coughs habitually at six A. M. Alumina.

— lasts all day, sensation of choking or suffocation, cannot bear bed-covers near his mouth for fear of choking, worse in a warm room; face gets deathly pale; cannot move, must sit perfectly still; chokes in sleep, which wakens him; worse lying on left side. Amm-c.

— with coldness between scapula and shoulders. Amm-m.

— child grasps throat with each cough. Anti-tart., Cepa, Acon.

— every effort to, starts the tears; expectoration of a soft brick-shade, quite tough, falls in jelly-like lumps; better lying on painful side; worse on back. Bry.

— better after breakfast; worse lying on right side (more from right lung); suffocating and choking at five A. M., as if from dryness of larynx. Kali-c.

— worse talking, laughing, walking, and deep inspiration, with painful roughness and constriction. Mang-acet.

— in winter, returns every winter. Psor.

— with early morning diarrhœa (Sulph.); cross and irritable (Cham.); peevish; worse from hurried, deep inspiration, speaking, and pressure on trachea. Rumex.

Cough dry and hard, constant desire to clear the throat; seems filled up, but cannot raise anything; oppression through chest; nausea from stomach during and after. Sepia.

— principally at night, with retching; comes in rapid concussions till breath is exhausted; then gagging and vomiting of mucus; worse before twelve at night; croup-like. Sepia.

— constant when child is laid down. Sepia.

— drinking cold water always brings on a severe cough; patient is quiet with the cough. Squilla.

— on deep inspiration, with cutting pain in left chest; great dyspnœa. Sulph.

— till completely exhausted, and then a cold perspiration on forehead. Verat-alb.

Whooping-cough, with much sneezing, watering of eyes and nose; child rubs eyes with hand. Squilla.

— — child stiffens out, muscles become rigid, and a clucking sound as the child comes out of the paroxysm. Cina.

— — in the beginning of the attack the coughs follow so closely, almost run into one another. Coral-rub.

— — from excessive secretion of mucus in trachea; from loud speaking; the slightest movement toward laughing after eating, with retching and vomiting of what has been eaten; *only in the daytime.* Dulcamara.

### Respiration.

Dyspnœa on falling asleep. Amm-c., Anti-tart., Arum-tri., Badiaga, Bry., Cadm-s., Carb-an., Carb-v., Grindel-robust., Graph., Laches., Nux-m., Op., Ranunc-bulb., Grindelia-sqarros.

— due to chronic aortitis. Oxal-ac., Spigel.

— gasping for breath immediately on sitting up. Laurocer.

Expiration difficult. Chlorine.

Inspiration — . China, Ferr., Nux-vom., Phos., Sambucus-nig.

Asthma. Camph., China, Natr-sulph., Sepia, Silphium-lac.

— from moving arms with force or from stooping. Amm-m.

— worse in cold weather. Apis.

— alternating with rash on chest. Caladium.

Asthma. Fear of going to sleep on account of losing his breath, which awakens him. Grindel-robust.

— awakens with attacks of, greenish purulent expectoration and loose evacuations immediately after rising. Natr-sulph.

— with suffocative attacks, they sleep into them. Sambuc-nig.

— spasmodic, with high-pitched voice. Stram.

Respiratory troubles, in, wants doors and windows open. Sulph.

Breathing slow and difficult, chest feels constricted. Laches.

Dyspnœa, sits up at night and opens window for fresh air. Plumb.

Puffing noise with breathing, in labor or after loss of fluids. China.

Breathing anxious, difficult, is obliged to get up and open window, better from fresh air, but immediately on lying down, the sensations return, as if ants were running through whole body; anxious, difficult breathing. Cann-ind.

Dyspnœa, it seems as if he cannot survive for want of air, has to be fanned to be kept alive. Apis.

Air, warm, cannot endure, must have doors and windows open. Amyl-nitros.

Air, fresh, desire for, or to be fanned. Apis, Cannab-ind., Carb-v., China, Cistus, Plumb., Puls., Sulph.

### Chest and Heart.

Beating, patient thinks the heart would stop beating, if he dared to move. Digit.

Move, patient thinks he must move in order to keep the heart from ceasing to beat. Gels.

Ball, sensation of a round ball, going to and fro under the ribs. Cupr.

Hydropericardium, from rheumatic pericarditis. Stictapulm.

Oppression of chest, panting respiration and palpitation, trembling and thumping of heart, as from fright, constant desire for fresh air, heart suddenly ceases to beat and feels as if squeezed or compressed. Tarent.

Oppression of chest, chilliness, thirstlessness. Puls.

Heart seems to be suddenly pulled up and then let go again, when going to sleep, which startles her. Magnes-carb.

Palpitation, not painful, after quick or violent motion. Phos. Silicea, Spigel.

— after every motion. Phos.

— from slightest motion. Spigel.

— — moving arms. Digit.

— with heat rising from pit of stomach upward, from bodily exertion. Ferr.

— from raising arms. Digit., Ledum., Nux-m., Nux-v., Plumb., Puls., Ranunc-bulb., Viola-tri.

Pain shooting from lower left chest to left shoulder. Sang.

Sensation under mid-sternum, like a lump of hot lead as big as two fists. Nux-vom.

Pneumonia, —after— lungs seem full of smoke; they smell pine smoke as if wood was burning. Baryta-c.$^{cm}$ .

— — lungs seem full of smoke as of paper burning. Coffea.

Chest; rash on alternating with asthma. Caladium.

Breathe, cannot, or move on account of pain in the lungs. Bry.

From mammary region a drawing pain through to back, right or left side; the pain may shift from one side to the other after the remedy, but do not change the remedy. Crot-tig.

Heat rising from pit of stomach up to chest; anxiety in chest after exercise. Ferr-met.

Stitches tearing in left breast and left short ribs not better by breathing alternating with toothache. Kali-c.

Dryness in chest; cannot talk on account of it; with red face and heat all over body. Kali-c.

Congestion to head and chest and arms, with cold feet; is obliged to move them constantly. Lil-tig.

Lungs, pain in worse from pressure in inter-costal spaces. Phos.

Pain in chest worse from use of arms. Rhus-t.

Constricted, chest feels; breathing slow and difficult. Lachesis.

Heart, irritable; increase of beats on rising, palpitation and fluttering on rapid motion or going up-stairs, with sharp pains. Bry.

Heart, when going to sleep, heart seems to be suddenly pulled up and then lets go again, which startles her. Magn-c.

### Chest, Mammæ, and Nipples.

Putting child to breast causes toothache. China.

— — — — — discharges from the uterus. Silicea.

— — — — — sharp pain to back. Crot-tig.

— — — — — cramps in back or abdomen. Puls., Cham.

— — — — — causes pain in the other, which the child does not nurse. Borax.

Suppuration threatened. Sulph.

— to assist. Hep., Merc., Silicea.

Nursing, after; child habitually cries for water and always throws it up. Arnica.

Nipples painful, with bluish blisters and sticky discharge. Graph.

### Neck, Back, Sacrum, etc.

Beating and fullness in sides of neck, feels as if all the blood left the heart and went to the head. Cimicif.

Cold in small spots on back and epigastrium; remedies having similar symptoms. Asar., Cinchon., Glon., Hæmatox, Lachnanthes, Paris, Rhus-t., Spigel, Vespa.

Cracking in head. Bell., Carlsbad., Kalm., Digit., Sep.

— — cervical vertebræ on moving head. Aloe 8 A. M., Calc-c., Coccul., Natr-c., Niccol., Oleum-an., Puls., Stan., Nitric-acid, Nux-v., Thuja, Sulph., Petrol.

Crushing, gnawing pains, violent at base of brain or in upper spine as if a dog were gnawing there; drawing back of the neck and spasms of back; mental irritability, gloom, and delirium, sadness; congestion of blood to head. Natrum-sulph.

Pain in back, aching intensely and burning along whole spine, patient cannot sit at all. Kobalt., Puls., Sepia, Zinc.

— — — better from walking. Puls., Rhus, Sep., Zinc.

— *from left scapula,* through left side, to infra-mammary region. Laurocer.

— in back better from lying on back. Ruta.

Spine, injuries to. Arnica, Rhus-t., Calc-c., Hyperic., Nitr-ac.

Sacrum extremely tender. Lobel.

Torticollis. Phos. to be followed by Natr-mur., also, Asafœt., Bell., Calc., Iod., Merc., Mezer., Silicea.

— old neglected cases. Sulph.

— congenital. Brucea, Pinus-sylvest.

Coccygodynia; from fall or blow. Arn., Ham.

— — strain during labor. Rhus-tox.

— — gout or rheumatism. Acon., Bry., Colchic., Amm-m., Manaca, Acid-salycil, Lithium-carb., Rhus-t., Cimicifug., Caulophyl.

— neuralgic. Acon., Bell., Cann-ind., Zinc-valer.

— pains worse after sleep. Lachesis.

— after confinement; burning and smarting, painful uneasiness better from standing, worse from slightest motion or pressure. Tarentula.

— after confinement, at and during first appearance of menses. Cicuta.

Coccyx, periodic aching in. Fluor-ac., Rhus, Ruta, Silicea.

— pain in. Bell., Caust., Caulophyl, Cimicifug., Rhus-tox., Ruta, Thuja, (Cannabis-s., Canthar., Cicuta, Fluor-ac., Graph., Kali-carb., Kreos., Lachesis, Magn., Merc., Mur-ac., Paris, Petrol., Phos., Phos-ac., Plat., Silicea, Zinc., etc.)

— burning when touched. Carb-an.

— pressive sore pain in lower spine and coccyx. Carb-v.

— dull drawing bruised pain. Caust.

— tearing, jerking. Cicuta.

— — — better from motion. Rhus-t.

Coccyx, painful uneasiness and stiffness when sitting. Petrol.

— and small of back worse from sitting. Petrol.

— — sacrum, numbness in, when sitting. Plat.

— — back bruised pain. Ruta.

— painful as after a long carriage ride, stinging and painful to pressure. Silicea.

— and lumbar region, violent bruised pain in, especially worse stooping or rising from a seat. Sulph.

Coccyx and sacrum, painful drawing also in thighs, while sitting; after long sitting, prevents standing erect. Thuja.

— gnawing in. Kali-carb.

Nape of neck, pain in, on going to bed, ceases on rising A. M. Alumina.

Coccyx, pain in, when sitting or lying. Amm-m.

— heavy weight and dragging in. Antimon-tart.

Backache with nausea. Colocynth.

— — — and scanty urine. Kali-bichro.

Pain from spine to head and shoulders, with contraction of dorsal muscles. Gels.

Injuries to spine; profuse perspiration breaks out on hands and feet. Nitric-acid.

Sacrum to pubes, pain all the way from. Sabina.

Shoulder-blade, pain under left. Sepia (Bell., Chenopod.), Cimicif., China, Ars., Gels., Ailanth.

Neck, muscles of, rigid; head drawn firmly back; from use of tobacco; convulsions. Lycopod.

### Upper Extremities.

Axilla, boils in. Lycopodium.

— glands enlarged. Bell.

Gurgling in shoulder-joint, or sensation as of something alive, especially at midnight. Berb.

Hands feel twice the size in severe diseases when patient wakes up; he cannot use them. Arnanea-diadem.

Pain on inner side of left arm from elbow to hand; begins at 3 A. M. and is worse in forenoon. Thuja.

Perspiration offensive in arm-pit. Hep., Dulc., Nitr-ac., Rhodod., Selen., Sepia, Thuja, Tellur.

— salty, so much as to leave a white salty deposit on outer coat in axillary region. Natr-mur. (high).

Washing hands, continually. Syphilin.

Axilla, boils in, scurfy, itching, moist herpetic eruption; pus continues to discharge from boils; they are no sooner healed than fresh ones appear. Lycopod.

Limbs cold and heavy; hands and feet cold; thirstless. Gels.

Hands chap, are sore, and bleed from working in water; averse to cold and open air. Calc-c.

Knees, face, and limbs cold; knees cold in bed. Carb-veg.

Fingers, the use of scissors leaves deep marks on fingers. Bovist.

### Lower Extremities.

Bones of lower limbs inflamed. Merc-v., Phos., Puls., Ruta, Sil., Staph.

Broken; sensation as if bones are broken in middle of thigh on sitting down. Illicium-anisat.

Contraction and stiffness in hollow of knees, and dribbling of urine. Staph.

Cracking in knee-joints. Ars., Caps., Nitric-ac., Sepia.

Nates feel cold, objective and subjective. Agar-musc.$^{99m}$.

Elongated feeling of right leg at night on lying down. Carbo-an.

— — — left leg, and the leg is actually two inches longer, as a result of rheumatism and old-school treatment, probably due to calcareous deposits between the joints. Thuja cured.

Heel affected. Caust., Graph., Ignat., Natr-c., Puls., Sabina, Sepia.

Sole of foot affected. Cupr., Mur-ac., Phos-ac., Puls., Sulph., Tart-emet.

Lies with knees to chin. Laurocer.

Lift, cannot, one foot without the other. Veratr-vir.

Itching, ball of right great toe, when taking boot off at night. Natr-sulph.

Nails, like sticking in heel. Puls.

Perspiration of feet, profuse, corrosive, and stinking, destroying stocking and shoes. Secale.

Shortening feeling of left leg in walking. Cinnabar.

— — of right leg on rising. Caust.

Sharp, shooting pains in a broken leg from heels to hips; leg is jerked upward. Phytolacca.

On going down-hill, knees weak. Bell.

Limb, right, seems shorter. Ambr.

— left — —. Caust.

Soles of feet feel as if cushioned when walking. Apis.

Varicose veins in left leg below the knee, with excruciating pains; the slightest touch causes agony; worse where they are swollen in knots, they look as if they would rupture or ulcerate; unable to put feet to the ground; walking is impossible without great pain. Fluor-ac.

Limbs cold and heavy, hands and feet cold; thirstless. Gels.

— aching; useless; pain in left groin; falls fainting at menses. Magn-c.

Tibia, bluish nodes on; bones are sensitive to touch. Mang-acet.

Run forward, inclination to, if he tried to walk. Mang-acet.

Heel, pain in, like tacks or nails sticking in it. Puls.

Sciatica. Pain in hip and thigh intolerable when standing, as if thigh would break; better when putting feet upon a chair; worse when straightening out the limb. Valeriana.

— neuralgic or rheumatic pain from gluteal muscles or hip to knee, calf of leg or ankle; better after walking. Phos-ac.

— right or left limb; worse from slightest motion, yet can't lie still, though motion increases the pain; constant moaning; heaviness; better lying on affected side. Bry.

— from ankle to hip, must move constantly; better walking or shifting feet; worse nights; very sensitive to touch as if nerve were uncovered. Bell.

— left hip and thigh; inner side and right thigh; worse four P. M.; left knee, calf, foot, very violent; tearing, shooting, boring; worse from heat; pressure, flexing leg on abdomen; worse from coughing, sneezing, and pressing at stool; constipation; better at night, and keeping perfectly quiet; sensation of contraction, shortening, tearing, drawing, cramping in right calf and inner side of left thigh; all worse in daytime; better at night. Colocynth.

— left hip and thigh, groin and foot; worse at night; burning pains, anguish; better from heat. Ars.

— crampy pain; heavy weight; loss of voluntary motion; worse from motion and walking, at night. Gels.

Sciatica tearing from hip to feet and left big toe. Kalmia.

— left leg and hip; crampy, tearing, or bruised pain, drawing from left hypochrondria down through abdomen into testes; worse in evening, lying down, especially on left side and painful side; worse in motion, sitting on hard chair; worse from motion; sharp, lancinating pains; worse nights, awakened by the pains, has to turn on right side before he can rise. Kali-carb.

— tearing in right thigh and knee; awakens at night, worse nights, lying on affected side, and back; cannot remain in bed. Kali-iod.

— left side, pain as from a hot iron, worse after sleep, rising and sitting up, much better lying quietly in bed. Lachesis.

— left side, from great trochanter to calf, worse A. M. walking and bending leg, better sitting, lying, standing, or pressure. Kali-bichro.

— tearing in hip and knee-joint worse nights with pulsating pains, sore to touch, worse in warm bed. Merc-viv.

— involuntary limping, pains most in knee, worse from over-exertion and at night; dull aching, worse rising after sitting, cold damp weather, or sweating; continued motion, better from rubbing and heat, if warmed by exercise; tearing pains, numbness, formication, paroxysmal, better from warmth or sweating, better from continued motion. Rhus-tox.

